# Supplementary material for: SARS-CoV-2 Consensus-Sequence and Matching Overlapping Peptides Design for COVID19 Immune Studies and Vaccine Development
Source: Vaccines (Basel). 2020 Aug 6;8(3):444. doi: 10.3390/vaccines8030444 (PMC7565482; doi:10.3390/vaccines8030444)
Supplement: Supplementary file 1 [file vaccines-08-00444-s001.zip › vaccines-859333 sup5/Design of CoV2 OLP - Figure S1_resubmission.pdf]

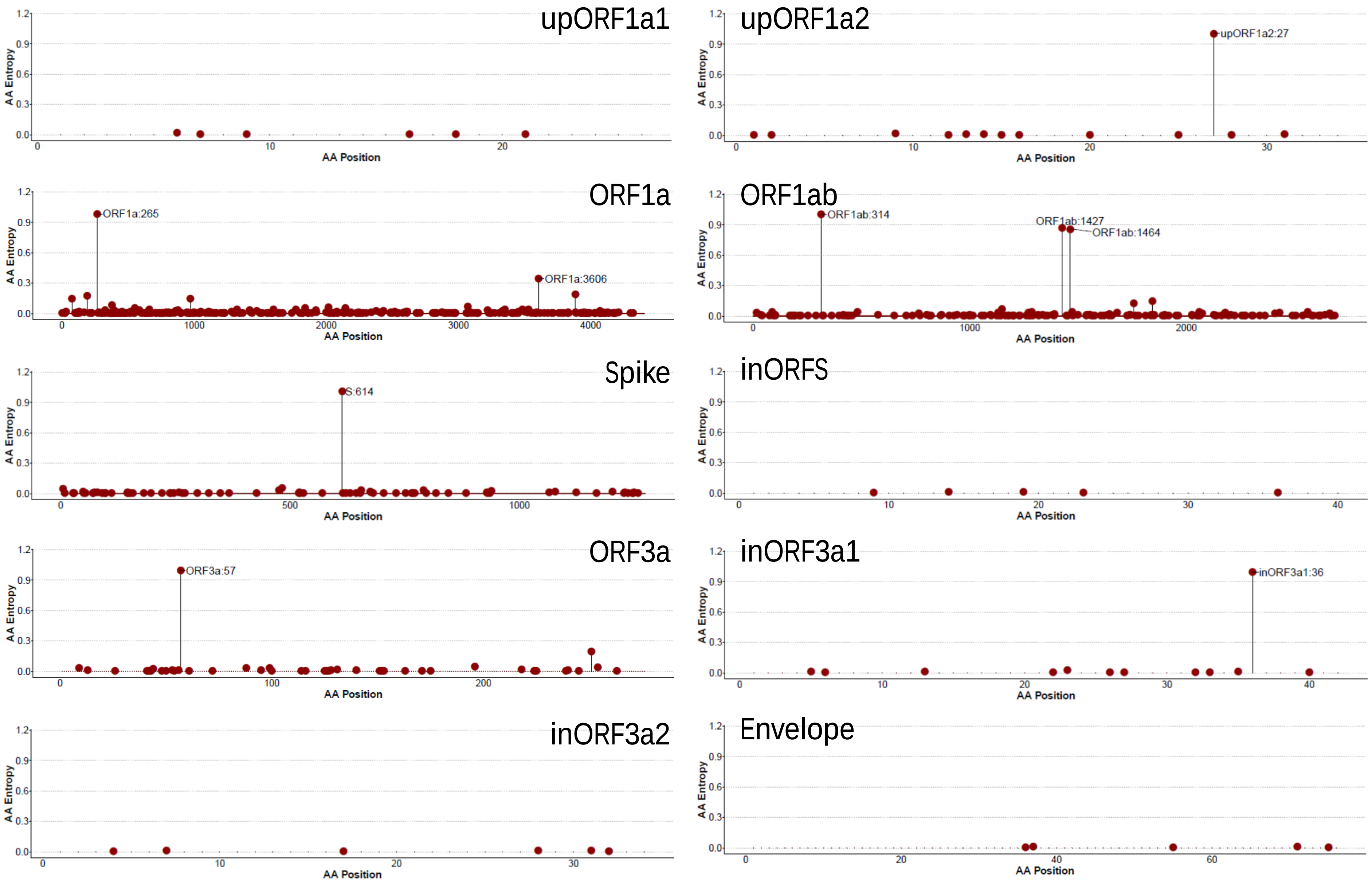
upORF1a1
upORF1a2
upORF1a2:27
ORF1a
ORF1a:265
ORF1a:3606
ORF1ab
ORF1ab:314
ORF1ab:1427
ORF1ab:1464
Spike
S:614
inORFS
ORF3a
ORF3a:57
inORF3a1
inORF3a1:36
inORF3a2
Envelope
AA Entropy
AA Position

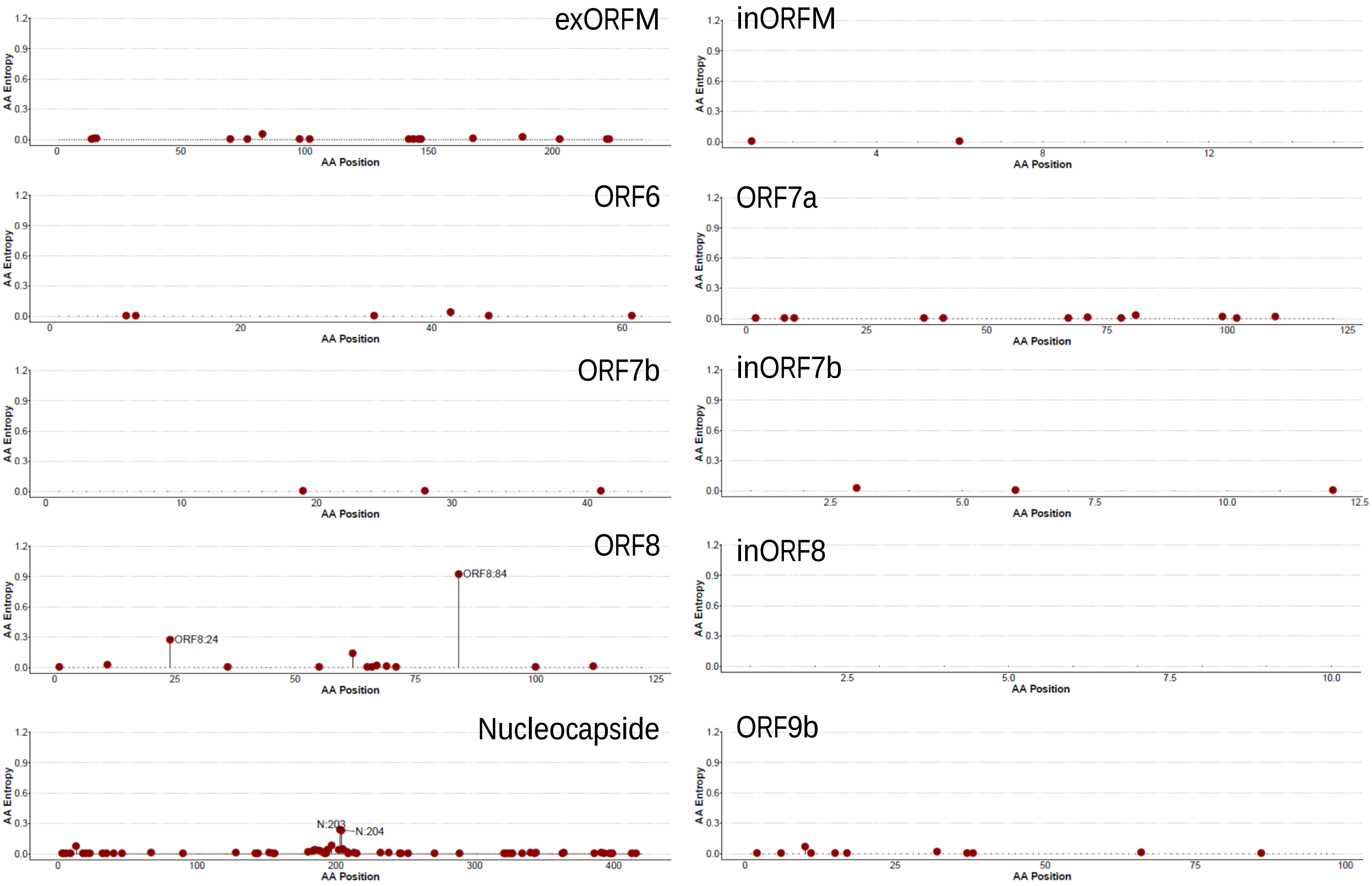
exORFM
inORFM
ORF6
ORF7a
ORF7b
inORF7b
ORF8
inORF8
Nucleocapside
ORF9b
AA Entropy
AA Position
ORF8:84
ORF8:24
N:203
N:204

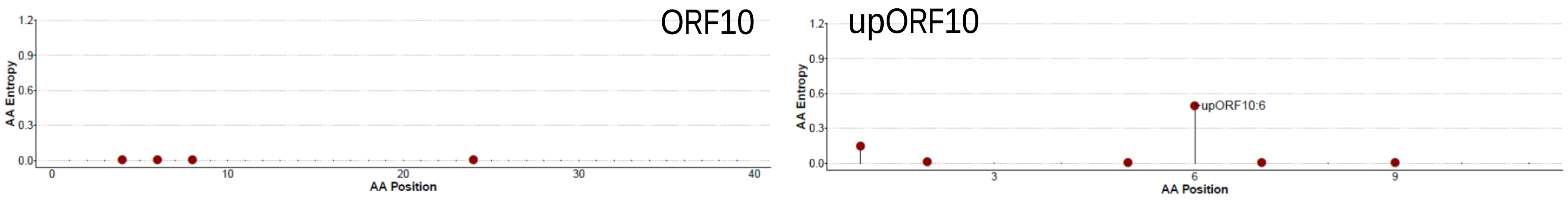

Supplementary figure S1. Shannon entropy plot by amino acid position for all canonical and alternative frame ORF of SARS-CoV-2. Only plot for the membrane protein is not included since it is completely embedded in exORFM.
