# Supplementary material for: SARS-CoV-2 Consensus-Sequence and Matching Overlapping Peptides Design for COVID19 Immune Studies and Vaccine Development
Source: Vaccines (Basel). 2020 Aug 6;8(3):444. doi: 10.3390/vaccines8030444 (PMC7565482; doi:10.3390/vaccines8030444)
Supplement: Supplementary file 1 [file vaccines-08-00444-s001.zip › vaccines-859333 sup5/Design of Cov2 OLP - Figure S2.pdf]

ORF1ab

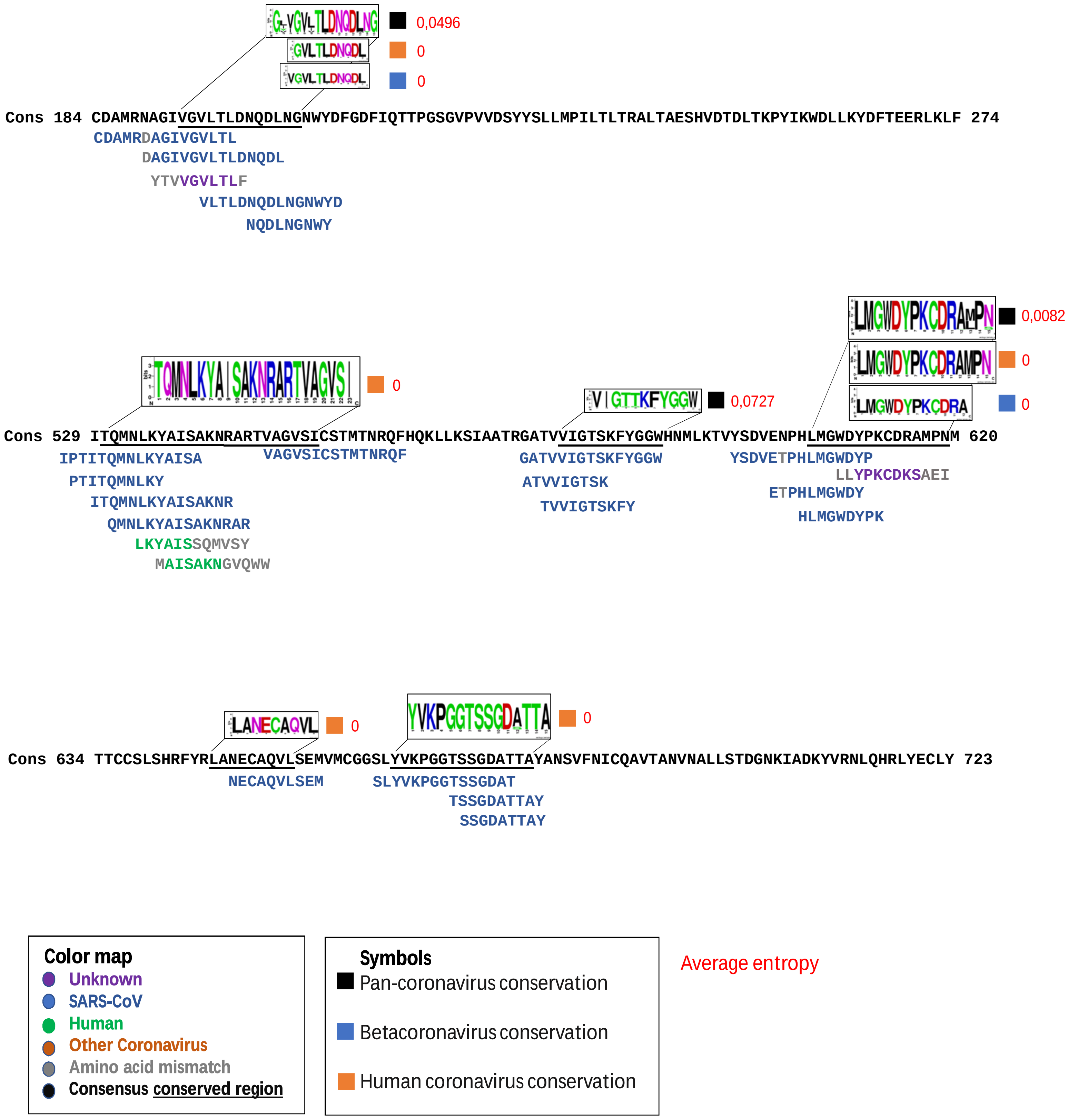
0,0496
0
0
Cons 184 CDAMRNAGIVGVLTLDNQDLNGNWYDFGDFIQTTPGSGVPVVDSYYSLLMPILTLTRALTAESHVDTDLTKPYIKWDLLKYDFTEERLKLF 274
CDAMRDAGIVGVLTL
DAGIVGVLTLDNQDL
YTVVGVLTLF
VLTLDNQDLNGNWYD
NQDLNGNWY
0,0082
0
0
0
0,0727
Cons 529 ITQMNLKYAISAKNRARTVAGVSICSTMTNRQFHQKLLKSIAATRGATVVIGTSKFYGGWHNMLKTVYSDVENPHLMGWDYPKCDRAMPNM 620
IPTITQMNLKYAISA
VAGVSICSTMTNRQF
PTITQMNLKY
ITQMNLKYAISAKNR
QMNLKYAISAKNRAR
LKYAISSQMVSY
MAISAKNGVQWW
GATVVIGTSKFYGGW
ATVVIGTSK
TVVIGTSKFY
YSDVETPHLMGWDYP
LLYPKCDKSAEI
ETPHLMGWDY
HLMGWDYPK
0
0
Cons 634 TTCCSLSHRFYRLANECAQVLSEMVMCGGSLYVKPGGTSSGDATTAYANSVFNICQAVTANVNALLSTDGNKIADKYVRNLQHRLYECLY 723
NECAQVLSEM
SLYVKPGGTSSGDAT
TSSGDATTAY
SSGDATTAY
Color map
Unknown
SARS-CoV
Human
Other Coronavirus
Amino acid mismatch
Consensus conserved region
Symbols
Pan-coronavirus conservation
Betacoronavirus conservation
Human coronavirus conservation
Average entropy

ORF1ab

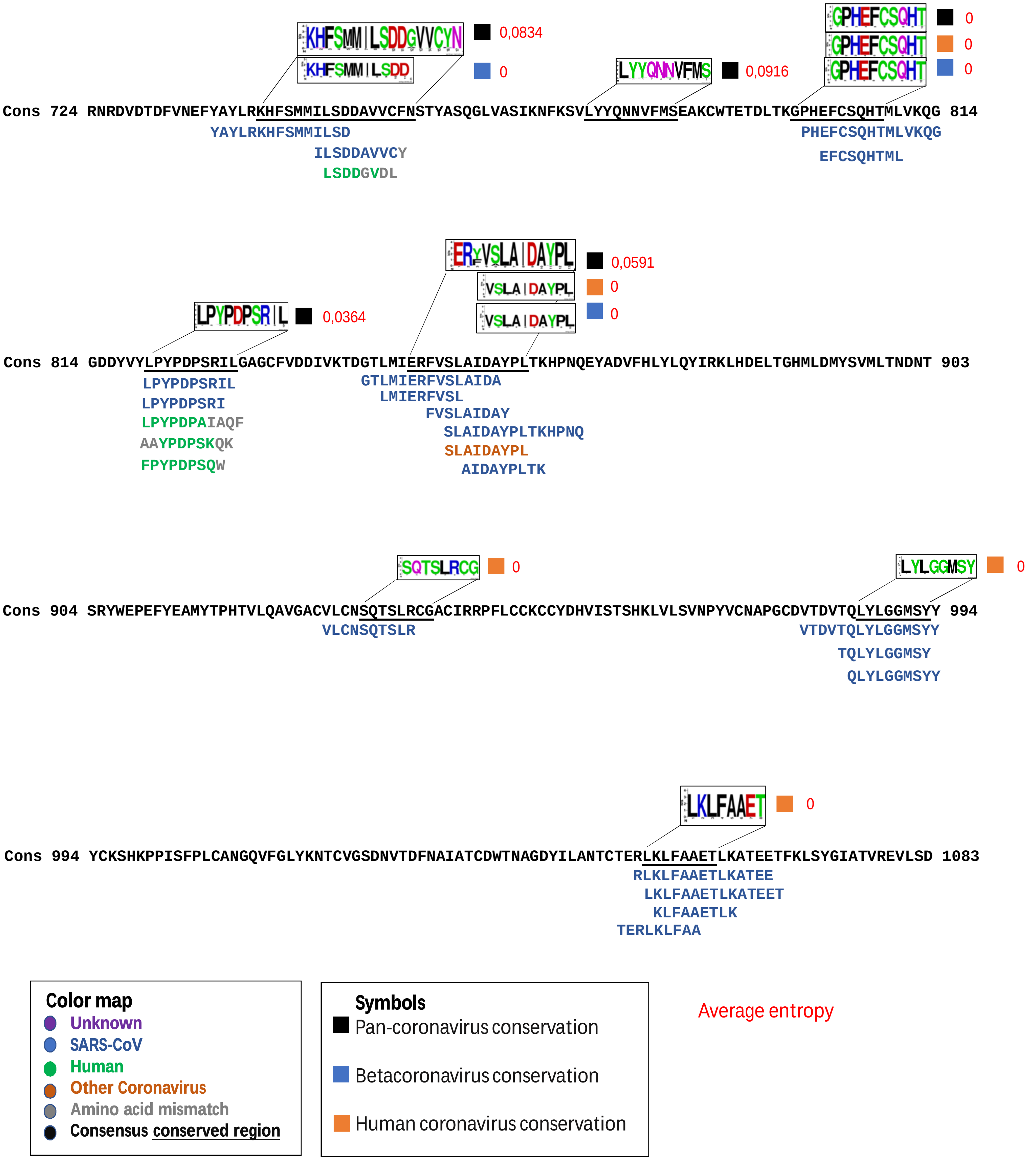
KHFSMMILSDDGVVCYN
0,0834
KHFSMMILSDD
0
LYYQNNVFMS
0,0916
GPHEFCSQHT
0
GPHEFCSQHT
0
GPHEFCSQHT
0
Cons 724 RNRDVDTDFVNEFYAYLRKHFSMMILSDDAVVCFNSTYASQGLVASIKNFKSVLYYQNNVFMSEAKCWTETDLTKGPHEFCSQHTMLVKQG 814
YAYLRKHFSMMILSD
ILSDDAVVCY
LSDDGVDL
PHEFCSQHTMLVKQG
EFCSQHTML
ERFVSLAIDAYPL
0,0591
VSLAIDAYPL
0
VSLAIDAYPL
0
LPYPDPSRIL
0,0364
Cons 814 GDDYVYLPYPDPSRILGAGCFVDDIVKTDGTLMIERFVSLAIDAYPLTKHPNQEYADVFHLYLQYIRKLHDELTGHMLDMYSVMLTNDNT 903
LPYPDPSRIL
LPYPDPSRI
LPYPDPAIAQF
AAYPDPSKQK
FPYPDPSQW
GTLMIERFVSLAIDA
LMIERFVSL
FVSLAIDAY
SLAIDAYPLTKHPNQ
SLAIDAYPL
AIDAYPLTK
SQTSLRCG
0
LYLGGMSY
0
Cons 904 SRYWEPEFYEAMYTPHTVLQAVGACVLCNSQTSLRCGACIRRPFLCCKCCYDHVISTSHKLVLSVNPYVCNAPGCDVTDVTQLYLGGMSYY 994
VLCNSQTSLR
VTDVTQLYLGGMSYY
TQLYLGGMSY
QLYLGGMSYY
LKLFAAET
0
Cons 994 YCKSHKPPISFPLCANGQVFGLYKNTCVGSDNVTDFNAIATCDWTNAGDYILANTCTERLKLFAAETLKATEETFKLSYGIATVREVLSD 1083
RLKLFAAETLKATEE
LKLFAAETLKATEET
KLFAAETLK
TERLKLFAA
Color map
Unknown
SARS-CoV
Human
Other Coronavirus
Amino acid mismatch
Consensus conserved region
Symbols
Pan-coronavirus conservation
Betacoronavirus conservation
Human coronavirus conservation
Average entropy

ORF1ab

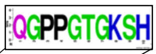

0

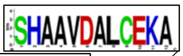

0

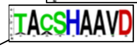

0,1287

```
Cons 1174 GLYPTLNISDEFSSNVANYQKVGMQKYSTLQGPPGTGKSHFAIGLALYYPSARIVYTACSHAAVDALCEKALKYLPIDKCSRIIPARARV 1263
```

RAVLLAGPPGTGKTALA

ALCEKALKY

GLLLYGPPGTGK

GVILYGPPGTGK

GILLYGPPGTGK

GVLLYGPPGTGK

LLYGPPGTGKTLLARAV

LLYGPPGTGKTLLAK

ILYGPPGTGKTLLAK

LLYGPPGTGKTLIARAV

ILYGPPGTGKTL

ILYGPPGTGKTLL

ILYGPPGTGK

LLYGPPGTGK

TLQGPPGTGK

LLFGPPGTGKSY

IIAGPPGTGK

ILFGPPGTGK

LLFGPPGTGK

LMYGPPGTGK

LMYGPPGTGK

LLAGPPGTGK

IIFGPPGTGK

MVGPPGTGK

LVGPPGTGK

IQGPPGTGKTY

LQGPPGTGK

IQGPPGTGK

LTGPPGTGK

VVGPPGTGK

MVGPPGTGK

YGPPGTGKTL

VGPPGTGKTL

FGPPGTGKTL

GPPGTGKSYLAKAVATEAN

GPPGTGKTLIARAVANETG

GPPGTGKTLIARAVANETGA

GPPGTGKTLIARAVANETGAF

GPPGTGKTAL

GPPGTGKTLL

GPPGTGKTLI

GPPGTGKTL

GPPGTGKTI

VPGTGKSTL

ORF1ab

GDPAQLPAPR ■ 0

```
Cons 1264 ECFDKFKVNSTLEQYVFCTVNALPETTADIVVFDEISMATNYDLSVVNARLRAKHYVYIGDPAQLPAPRTLLTKGTLEPEYFNSVCRLMK 1353
                                                                            HYVYIGDPAQLPAPR
                                                                                    PAQLPAPRTLLTKGT
                                                                              VYIGDPAQL
```

AVFISPYNSQN ■ 0,1113
AVFISPYNSQN ■ 0
AVFISPYNSQN ■ 0

NRFNVAITRA ■ 0

```
Cons 1429 WRKAVFISPYNSQNAVASKILGLPTQTVDSSQGSEXDYVIFTQTTETAHSCNVNRFNVAITRAKVGILCIMSDRDLYDKLQFTSLEIPRR 1518
            RKAVFISPY                                         VNRFNVAITRAKIGI
                FISPYTQDR                                       RFNVAITRAK
                 ISPYNSQNAVASKIL
                  SPYNSQNAVA
                  SPYNSQNAV
```

CNLGGAVC ■ 0

```
Cons 1984 VSDIDYVPLKSATCITRCNLGGAVCRHHANEYRLYLDAYNMMISAGFSLWVYKQFDTYNLWNTFTRLQSLENVAFNVVNKGHFDGQQGEV 2073
                         RCNLGGAVCRHHANE
```

KYTQLCQYLN ■ 0,1056

```
Cons 2434 PKGIMMNVAKYTQLCQYLNTLTLAVPYN 2461
          PKGIMMNVAKYTQLC
                   KYTQLCQYL
                       LCQYLNTLTLAVPYN
```

Color map
- Unknown
- SARS-CoV
- Human
- Other Coronavirus
- Amino acid mismatch
- Consensus conserved region

Symbols
- ■ Pan-coronavirus conservation
- ■ Betacoronavirus conservation
- ■ Human coronavirus conservation

Average entropy

Spike

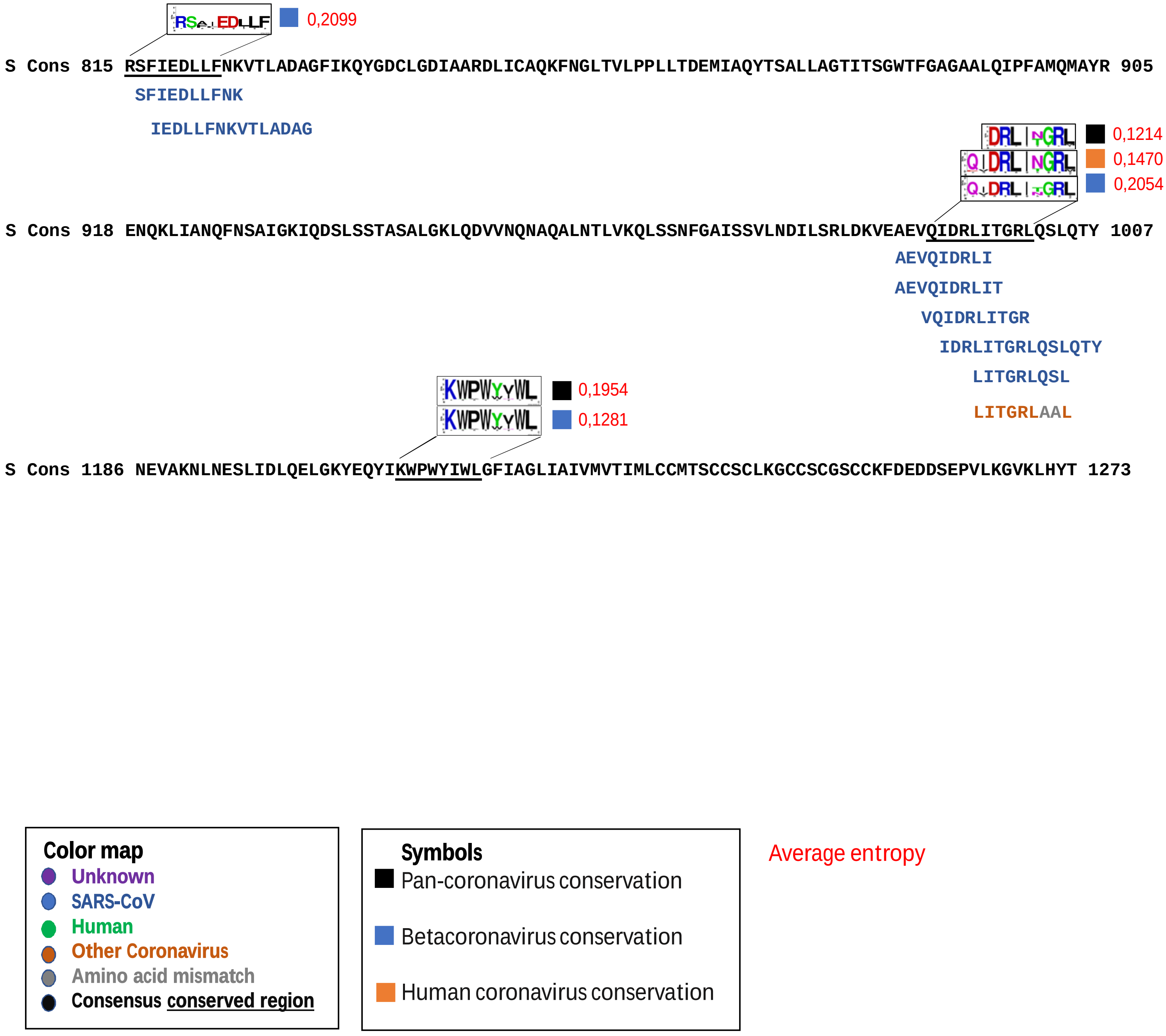
0,2099
S Cons 815 RSFIEDLLFNKVTLADAGFIKQYGDCLGDIAARDLICAQKFNGLTVLPPLLTDEMIAQYTSALLAGTITSGWTFGAGAALQIPFAMQMAYR 905
SFIEDLLFNK
IEDLLFNKVTLADAG
0,1214
0,1470
0,2054
S Cons 918 ENQKLIANQFNSAIGKIQDSLSSTASALGKLQDVVNQNAQALNTLVKQLSSNFGAISSVLNDILSRLDKVEAEVQIDRLITGRLQSLQTY 1007
AEVQIDRLI
AEVQIDRLIT
VQIDRLITGR
IDRLITGRLQSLQTY
LITGRLQSL
LITGRLAAL
0,1954
0,1281
S Cons 1186 NEVAKNLNESLIDLQELGKYEQYIKWPWYIWLGFIAGLIAIVMVTIMLCCMTSCCSCLKGCCSCGSCCKFDEDDSEPVLKGVKLHYT 1273
Color map
Unknown
SARS-CoV
Human
Other Coronavirus
Amino acid mismatch
Consensus conserved region
Symbols
Pan-coronavirus conservation
Betacoronavirus conservation
Human coronavirus conservation
Average entropy

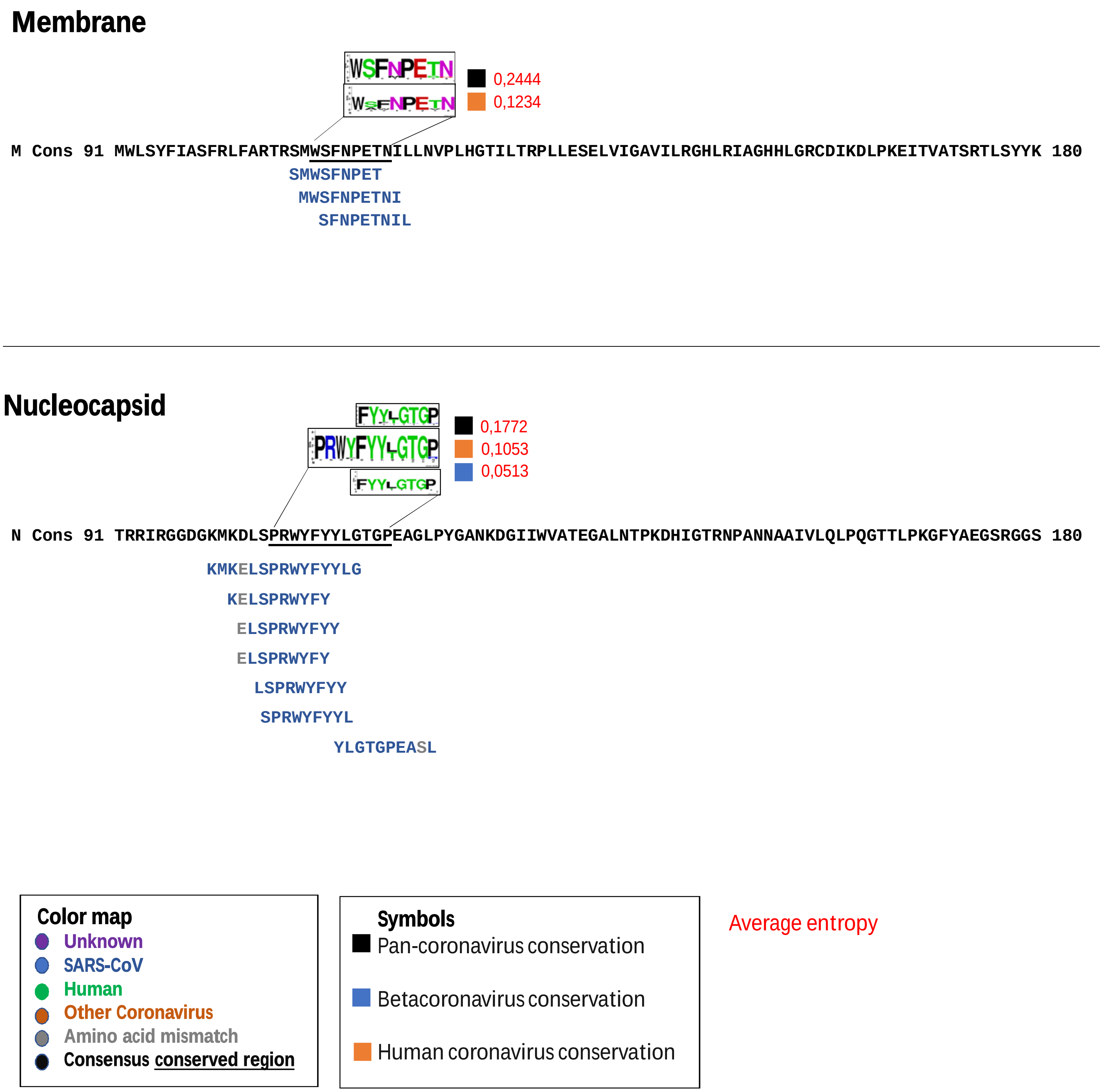

Supplementary Figure S2. SARS-CoV-2 ORF fragments containing conserved regions in Human, Betacoronavirus or Pan-coronavirus alignments are shown. Epitope description was carried out based on experimentally described epitopes from the Immune Epitope Database (IEDB, www.iedb.org). Species were the epitope was described are indicated by sequence color. Logos of conserved sequences along the three alignments (Pan-coronavirus, Betacoronavirus or Human coronavirus) were included. The alignment were the conserved regions were identified is indicated by color squares
